# Hospitalization costs in patients with stroke in southeastern China: a retrospective population-based cohort study, 2019–2022

**DOI:** 10.3389/fpubh.2024.1442171

**Published:** 2024-11-08

**Authors:** Jing Xu, Ruixue Ye, Jingpu Zhao, Xuehui Fan, Kaiwen Xue, Xiaoxuan Li, Xiaolong Zhu, Yan Gao, Yulong Wang

**Affiliations:** ^1^Department of Rehabilitation, Shenzhen Second People’s Hospital, The First Affiliated Hospital, Shenzhen University School of Medicine, Shenzhen, Guangdong, China; ^2^Rehabilitation Medical College, Shandong University of Traditional Chinese Medicine, Jinan, China

**Keywords:** stroke subtype, costs and cost analysis, inpatients, retrospective cohort study, southeastern China

## Abstract

**Introduction:**

Stroke remains a predominant cause of mortality and accounts for one-third of all stroke-related fatalities worldwide. Increasing expenses associated with stroke are a matter of significant concern; however, this aspect has been insufficiently examined.

**Methods:**

The purpose of this study was to analyze in-hospital stroke costs and explore potential factors influencing them across stroke subtypes. The records of stroke patients from 50 hospitals in southeastern China between 2019 and 2022 were reviewed using multistage stratified cluster random sampling. We focused on the cost patterns of four stroke types and used multivariate linear regression to identify cost determinants.

**Results:**

A total of 417 (1.1%) patients had subarachnoid hemorrhage (SAH), 9309 (25.9%) had intracerebral hemorrhage (ICH), 22,248 (61.8%) had ischemic stroke (IS), and 4025 had transient ischemic attack (TIA). The number of stroke patients has sharply increased since the onset of COVID-19, with a majority of them being male (72.2%). Despite the fact that hospitalization costs are highest in tertiary hospitals (Chinese yuan [CNY] 30610.8/United States dollar [USD] 4551.0, interquartile range [IQR] 9944.9, 29668.4/1478.6, 4410.9), the majority of patients are admitted to tertiary hospitals (74.6%) or public hospitals (90.2%). Across all stroke subtypes, patients with SAH had the highest costs (CNY 93,454.9/USD13894.4, IQR 12273.2, 169920.0/1824.7, 25262.8), followed by those with ICH (CNY 48,724.2/USD 7244.0, IQR 16789.6, 57540.7/2496.2, 8554.8), IS (CNY 26,550.3/USD3947.4, IQR 8684.2, 28697.7/1291.1, 4266.6), and TIA (CNY 11,170.1/USD1660.7, IQR 6823.7, 12965.2/1014.5, 1927.6). Therapy fees comprised a significant portion of costs in ICH and IS cases (47.9% and 42.7%, respectively). Materials accounted for the highest proportion of expenses for SAH (56.1%), whereas patients with TIA spent more time on examinations (34.1%). Linear regression analysis revealed that length of stay (LOS), stroke subtype, hospital level, and stroke type were key factors influencing hospitalization costs

**Discussion:**

The visiting rate and charges were highest in tertiary public hospitals, and hospitalization costs were higher in hemorrhagic types of stroke than in ischemic types of stroke; the proportion of hospitalization cost categories varied among different types of stroke, with LOS, hospital type, and level substantially affecting hospitalization costs. Enhancing medical insurance reimbursement rates for hemorrhagic strokes, implementing a hierarchical medical system, tailoring cost categories to accommodate varying stroke subtypes, and shortening LOS may help alleviate the economic burden of stroke.

## 1 Introduction

Stroke presents a rapidly growing public health challenge, exerting a substantial burden on society. In 2019, it stood as the second leading cause of both mortality (claiming 6.6 million lives) and long-term disability (accounting for 143 million disability-adjusted life years [DALYs]) globally. Over the last 30 years, there has been a 70% increase in global stroke incidence, an 85% rise in prevalence, a 43% spike in mortality, and a 32% surge in stroke-related DALYs. This growing burden is more pronounced in low-and middle-income countries compared to high-income nations (1). In China, despite improved public health policies and healthcare delivery, the burden of stroke remains alarmingly high, with a pooled annual prevalence of 1329.5 per 100,000 people based on a review of 26 population-based studies (2). The burden of stroke is anticipated to grow, leading to significant economic costs related to treatment and post-stroke care.

In recent years, hospitalization costs for patients with stroke have gained increased attention owing to their significant impact on healthcare systems and individual patients. The increasing prevalence of stroke, coupled with the complex and often long-term nature of stroke care, has contributed to the financial burden experienced by patients and their families. Additionally, the costs associated with acute treatment, rehabilitation, and post-stroke care further compound the economic challenges faced by stroke survivors.

In various Chinese cities, hospital costs for stroke treatment have been estimated in previous studies (3–6). However, findings from large comprehensive hospitals in northeastern and southwestern China, as well as nationwide assessments, may not be applicable to medical institutions in southeastern regions like Shenzhen city. There may be certain cost differences among medical institutions across different regions in China depending on factors such as the size of the hospital, its equipment, service level, and the economic development of the region (7). Additionally, different stroke types exhibit a distinctive disease progression and treatment procedures, resulting in varied medical resource usage and costs (1, 8). While several previous studies have estimated the direct burden of stroke treatment, they often concentrate on a single stroke type or utilize data from a single hospital, thus lacking broad representativeness (9). A thorough examination providing a comprehensive overview of healthcare utilization and expenditure in specific stroke service categories among Chinese patients is still lacking. However, understanding the economic burden distribution associated with different types of stroke is crucial for developing specific strategies to targeted prevention and control of disease.

Despite the considerable resources allocated to stroke treatment, many questions remain regarding the allocation of funds for different types of stroke conditions and how spending varies based on patient characteristics. Gaining insight into the variations in healthcare expenditure can assist health system researchers and policymakers in pinpointing the specific conditions, age groups, genders, and types of care that contribute to increased spending. This understanding can serve as a foundation for anticipating future economic challenges amid the rising incidence and prevalence of stroke, and can also guide the identification of areas where new technologies and processes might yield significant returns on investment.

To address this knowledge gap, this study aims to estimate stroke-related hospitalization costs using representative data from Shenzhen, southeastern China. Based on a retrospective analysis of real-world data on patient levels from 50 hospitals in Shenzhen from 2019 to 2022, we further evaluated the relationships between various sociodemographic factors, hospital attributes, disease characteristics, and stroke-related economic burdens to provide a descriptive summary of hospitals cost estimates for stroke.

## 2 Methods

### 2.1 Study design and population

We conducted a retrospective, population-based longitudinal cohort study in Shenzhen, China, from January 30, 2019 to December 30, 2022, on patients aged 1–108 with primary diagnosis of stroke. Medical institutions providing rehabilitation services in Shenzhen were included. The tenth revised version of the International Classification of Diseases (ICD-10) was utilized to identify patients diagnosed with stroke. Stroke stratification is as follows: ischemic stroke (IS), transient ischemic attack (TIA), subarachnoid hemorrhage (SAH), and cerebral hemorrhage (ICH) (10) using ICD-10 codes G45, I60, I61 and I63, respectively. Exclude stroke patients outside of IS, TIA, SAH and ICH. Further exclusion criteria encompassed hospital stays of less than 1 day, hospitalization costs below 100 Chinese yuan (CNY), or missing information. Data were obtained from medical records and hospital information systems. This study has been approved by the Shenzhen Health Security Bureau and follows the guidelines for reporting observational studies under the Strengthening Epidemiological Observation Research Report (STROBE).

### 2.2 Data source

**Figure 1:**
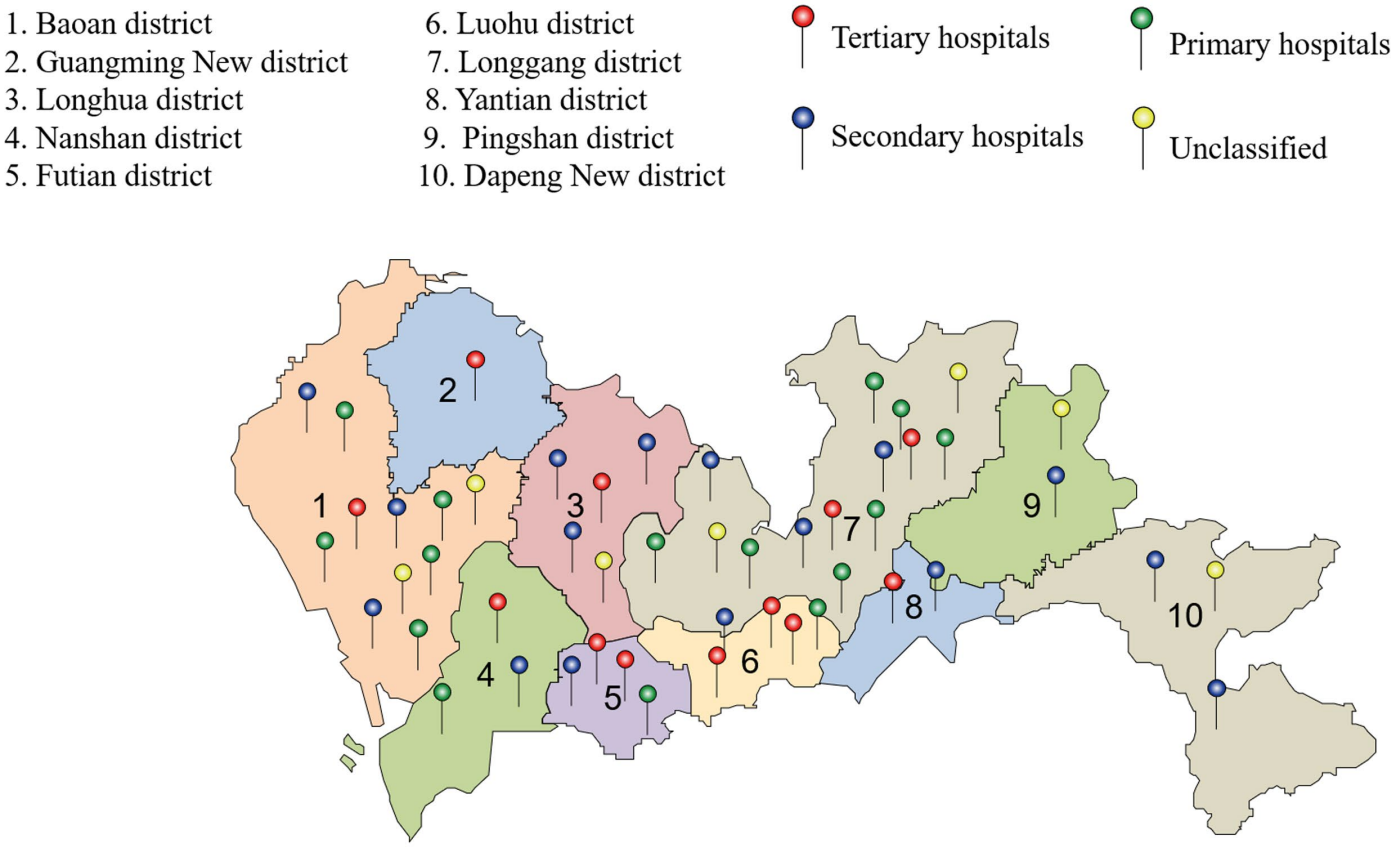
Locations of hospitals included in this study.

The data collection units included primary, secondary, and tertiary hospitals in Shenzhen as well as public and private hospitals, involving comprehensive disciplines, specialized departments, and rehabilitation hospitals covering the entire city. A total of 50 units were contacted during the initial stage (Figure 1). The database contained detailed information on the patient’s gender and age, initial diagnosis, hospitalization date, length of stay, and hospitalization expenditures/the total cost of hospitalization. Due to the anonymization of patient identity data, the local institutional review committee has abandoned the necessity of obtaining patient consent.

### 2.3 Measures

Study outcomes include the costs associated with hospitalization *per capita*, which are primarily based on stroke type, sociodemographic data, and typical characteristics of hospitals. The hospitalization expenses included prescribed medicines, rehabilitation therapy, surgeries, prescription drugs, imaging examinations, medical consumables, beds, laboratory investigations, diagnostic and nursing expenses. All costs were measured using continuous variables in CNY. The exchange rate between United States dollar (USD) and CNY was: USD1.00 = CNY 6.7261 in 2022. We also included the basic characteristics of the patients, including age, sex, profit status of medical institutions (private or public), level of medical institutions (unclassified, primary healthcare institutions, secondary hospitals, or tertiary hospitals), type of stroke (SAH, ICH, IS, or TIA), and study period (2019, 2020, 2021, 2022). The concepts of some measures are explained as follows:

1. Profit status of medical institutions: In China, hospitals can be categorized into two main types based on profit status: public (non-profit) and private (for-profit). Public hospitals are owned and operated by the government or state-owned enterprises, funded through government budgets, patient fees, and occasionally donations. In contrast, private hospitals are owned by individuals or corporations and aim to generate profits, with significant variation in size, specialization, and services offered. In our study, the profit status of hospitals was categorized as a dummy variable (0 = private; 1 = public).
2. Level of medical institutions: hospitals in China can be further classified based on size, function, level of care, etc. There are unclassified, primary, secondary, and tertiary hospitals. The unclassified hospitals usually were not equipped with enough hospital beds, staff, departments, and technology, which did not reach the standard of “Hospital Classification and Management Standards in China”. Primary hospitals directly provide basic medical services, including primary care, vaccinations, health education, and rehabilitation services to residents in certain communities. Secondary hospitals typically offer comprehensive medical services to multiple communities and undertake certain teaching and research tasks. Tertiary hospitals provide higher-level specialized medical and health services in several areas and perform higher teaching and research tasks. In our study, the level of hospitals was coded as a category variable (0 = unclassified hospitals, 1 = primary hospitals, 2 = secondary hospitals, 3 = tertiary hospitals).
3. Type of stroke: Each type of stroke has distinct clinical characteristics. SAH involves bleeding into the space between the brain and the tissues covering it, often due to the rupture of an aneurysm. ICH is characterized by the sudden accumulation of blood within the brain, often caused by hypertension or vascular malformations. IS occurs when a blood vessel supplying the brain is obstructed, resulting in tissue ischemia. TIA are temporary episodes of neurological dysfunction caused by transient ischemia.

### 2.4 Statistical analysis

Descriptive statistics were used to outline the characteristics of the patients, stroke types, and hospital features. Due to the fact that average cost provides a comprehensive overview of social burden and is crucial for economic analysis, we predominantly use means to present our research findings. However, owing to the typical positive bias nature of cost data, we also provided results for median and interquartile range (IQR) (11). In order to determine the significance of the observed cost differences, we first conducted a correlation analysis between various factors (see Supplemental Table 1) and univariate analyses using the Mann–Whitney rank-sum test. The cost data were logarithmically transformed to achieve a corrected distribution. The regression model was used to analyze the associated factors of hospitalization costs. The basis of the estimation method in this study is multilevel regression. As shown in Equation 1, i = 1, 2, …, n, indicates the unit of level 1 (patients), j = 1, 2, …, n, indicates the unit of level 2 (hospital). $Y_{ij}$ was the outcome from patients ‘i’ at hospital ‘j’. $\beta_{0j}$ is the intercept, which indicates the average estimate of outcome among ‘hospital j’ at baseline. $\mu_{0j}$ represents the difference between the average cost from ‘hospital j’ $\beta_{0j}$ and the average cost $\beta_{0}$ , which is the residual error in level 2, $\sigma_{\mu_{0}}^{2}\;\mathrm{is}$ the variance of $\mu_{0j}$ that can be used to estimate the difference between hospitals. The coefficient of ‘ $\beta_{1j}$ ’ is the slope, which is different between different hospitals and represents the slope of the change of $Y_{ij}$ with $X_{ij}$ from ‘hospital j’. $\mu_{1j}$ represents the difference between the average slope from ‘hospital j’ $\beta_{1j}$ and the average slope $\beta_{1}$ , which is also the residual error in level 2, $\sigma_{\mu_{1}}^{2}$ indicating the variance of the $\mu_{1j}$ . $X_{\mathrm{it}}$ was a series of independent variables on ‘patient i’ from ‘hospital j’. $\varepsilon_{0ij}$ is the random error term, which denotes the random error between different patients, $\sigma_{\varepsilon_{0}}^{2}$ is the variance of $\varepsilon_{0ij}$ that can be used to estimate the difference between patients. $\sigma_{\mu_{01}}$ refers to the covariance of difference between the intercept and slope, reflecting the correlation between the intercept and slope. To check multicollinearity, generalized variance-inflation factor (GVIF) (12) was employed and a GVIF value below 10 was considered acceptable (13). STATA 15.0 and SPSS 22.0 software were used for analysis, and we used *p* = 0.05 as the threshold for statistical significance.


(1) $$Y_{ij}=\beta_{0j}+\beta_{1j}X_{ij}+\varepsilon_{0ij}\text{,}\;\beta_{0j}=\beta_{0}+\mu_{0j}\text{,}\;\beta_{1j}=\beta_{1}+\mu_{1j}\text{,}\;\mu_{0j}\sim N\left(0,\sigma_{\mu_{0}}^{2}\right),\mu_{1j}\sim N\left(0,\sigma_{\mu_{1}}^{2}\right),\varepsilon_{0ij}\sim N\left(0,\sigma_{\varepsilon_{0}}^{2}\right)\text{,}\;COV\;\left(\beta_{0j},\beta_{1j}\right)=\sigma_{\mu_{01}}$$

## 3 Results

### 3.1 Patient characteristics

In total, we included 35,999 immobile stroke patients who required hospitalization between January 2019 and December 2022. Table 1 shows the profile of the patients’ sociodemographic and hospital characteristics of patients classified by stroke type. Among the assessed patients, 417 (1.1%) had SAH, 9309 (25.9%) had ICH, 22,248 (61.8%) had IS, and 4,025 (11.2%) had TIA. More than half of the patients were male (72.2%). The mean age of the patients was 59.4 (± 15.3) years, with the youngest being 1 year old. The average LOS was 25.1 (± 30.8) days, with the longest hospitalization for ICH at 35.3 (± 37.1) days and the shortest for TIA at 7.7 (± 6.2) days. Most patients were admitted to tertiary (74.6%) or public hospitals (90.2%). On average, patients who suffered from hemorrhagic strokes (SAH and ICH) were younger than those with TIA and IS. Among all groups, patients with cerebral infarctions were the oldest, with a mean age of 62.2 years. At 35.3 days, patients with ICH had the longest average hospital stay. Since 2020, the number of patients with SAH, ICH, IS, and TIA has significantly increased, with stroke cases in both 2021 and 2022 exceeding 10 times that of 2020.

**Table 1:** Socio-demographic and hospital characteristics of patients by type of stroke.

Variable	SAH	ICH	IS	TIA	Overall
N (%)	417 (1.1)	9,309 (25.9)	22,248 (61.8)	4,025 (11.2)	35,999 (100.0)
Gender, male, n (%)	253 (60.7)	7,269 (78.1)	16,448 (74.0)	2028 (50.4)	25,998 (72.2)
Age, years, mean (SD)	50.1 (14.0)	53.1 (14.6)	62.2 (15.1)	59.4 (13.6)	59.4 (15.3)
<45	133 (31.9)	2,377 (25.5)	2,365 (10.6)	463 (11.5)	5,338 (14.8)
45–64	240 (57.6)	5,061 (54.4)	10,795 (48.5)	2,267 (56.3)	18,363 (51.0)
>64	44 (10.5)	1871 (20.1)	9,088 (40.8)	1,295 (32.2)	12,298 (34.2)
LOS, days, mean (SD)	17.6 (18.4)	35.3 (37.1)	23.6 (28.8)	7.7 (6.2)	25.1 (30.8)
Surgery, n (%)	268 (64.3)	1885 (20.2)	2,605 (11.7)	1,035 (25.7)	5,793 (16.1)
Hospital level, n (%)					
Unclassified	7 (1.7)	255 (2.7)	1929 (8.7)	46 (1.1)	2,237 (6.2)
Primary hospital	11 (2.6)	793 (8.5)	1,343 (6.0)	110 (2.7)	2,257 (6.3)
Secondary hospital	24 (5.8)	1741 (18.7)	2,643 (11.9)	247 (6.1)	4,655 (12.9)
Tertiary hospital	375 (89.9)	6,520 (70.1)	16,333 (73.4)	3,622 (90.1)	26,850 (74.6)
Hospital type, n (%)					
Public hospital	397 (95.2)	7,780 (83.6)	20,387 (91.6)	3,909 (97.1)	32,473 (90.2)
Private hospital	20 (4.8)	1,529 (16.4)	1861 (8.4)	116 (2.9)	3,526 (9.8)
Admission year, n (%)					
2019	4 (1.0)	543 (5.8)	757 (3.4)	0 (0.0)	1,304 (3.6)
2020	8 (1.9)	583 (6.3)	759 (3.4)	0 (0.0)	1,350 (3.8)
2021	196 (47.0)	4,485 (48.2)	9,743 (43.8)	1,636 (40.6)	16,060 (44.6)
2022	209 (50.1)	3,698 (39.7)	10,989 (49.4)	2,389 (59.4)	17,285 (48.0)

SAH, subarachnoid hemorrhage; ICH, intracerebral hemorrhage; IS, ischemic stroke; TIA, transient ischemic attack; LOS, length of stay.

### 3.2 Hospitalization costs

The estimated average hospitalization cost for stroke patients was CNY 31,661.4/USD 4707.2 (see Table 2). Across all stroke subtypes, patients with SAH had the highest costs (CNY 93,454.9/USD 13894.4), followed by those with ICH (CNY 48,724.2/USD 7244.0), IS (CNY 26,550.3/USD 3947.4), and TIA (CNY 11,170.1/USD 1660.7). Univariate analysis revealed that higher costs were significantly linked to male sex, older age, admission to secondary, tertiary, or private hospitals, as well as earlier admission years (all *p* < 0.001).

**Table 2:** Hospitalization costs of patients by type of stroke, in Chinese Yuan (CNY).

	SAH	ICH	IS	TIA		Overall	
Variable					Mean	Median (IQR)	*p* value
Gender							<0.001
Male	87583.6	48366.8	26170.0	10824.5	31776.8	17613.8 (9396.1–33085.9)	
Female	102512.4	49983.1	27628.8	11521.0	30202.4	14922.9 (8266.2–31584.6)	
Age group							<0.001
<45	82101.3	50130.3	25892.4	9425.3	36657.7	19285.7 (9769.6–36991.3)	
45–64	107307.9	46328.2	23748.3	11568.1	29533.5	16389.9 (9377.6–31203.5)	
>64	61789.9	53433.9	30054.4	11108.2	31727.4	16538.3 (8221.4–33920.7)	
Hospital level							<0.001
Unclassified	16434.8	4222.1	4266.5	5687.7	4328.7	2556.3 (1250.1–5070.2)	
Primary hospital	12956.9	57158.6	42908.6	5839.3	45962.7	29224.0 (9466.3–73112.4)	
Secondary hospital	84028.4	52354.1	36865.6	9006.9	41432.0	30829.6 (11232.5–56822.6)	
Tertiary hospital	97857.1	48460.1	26168.1	11551.7	30610.8	16863.1 (9944.9–29668.4)	
Hospital type							<0.001
Public hospital	96802.3	44962.8	24014.9	11304.6	28401.3	15863.2 (8869.0–29739.7)	
Private hospital	27008.6	67692.5	54319.2	6701.0	58398.1	42636.1 (13776.2–88143.6)	
Admission year							<0.001
2019	77877.5	48435.5	42136.3	0	44869.0	28503.6 (18409.3–66978.7)	
2020	115274.9	54318.2	54270.5	0	54666.7	37223.7 (21653.8–74746.7)	
2021	96546.5	52304.3	27016.1	10773.0	33272.1	17546.3 (9088.4–34260.4)	
2022	90018.5	43541.2	23149.1	11447.3	26701.0	14515.0 (8431.1–28092.9)	
Total costs	93454.9	48724.2	26550.3	11170.1	31339.4	16831.0 (9064.7–32726.8)	

IQR, Interquartile range. Mann–Whitney rank-sum tests were used to determine the significance of observed differences in median costs.

After adjusting for these factors, multiple regression analysis showed that LOS, SAH, and admission to tertiary, private hospitals or 2022 were independently contributed to higher costs (see Table 3 and Supplemental Tables 2-5). Compared with patients with ICH, the mean hospitalization cost of patients with TIA was 9.7% lower, whereas the increase in hospitalization costs of patients with SAH (13.4%) was greater. As the hospital level increased in sophistication, the average hospitalization cost also increased. Tertiary hospitals bear the highest hospitalization expenses, with a significant increase of 26.3% compared to unclassified hospitals, and rises of 12.0% and 12.9% when compared to primary and secondary hospitals, respectively. Hospitalization expenses in public hospitals have seen a slight decrease of 0.3% compared to those in private hospitals. The average hospitalization cost for stroke patients has risen steadily over the years, with costs in 2022 increasing by 3.6%, 1.8%, and 1.0% compared to 2019, 2020, and 2021, respectively.

**Table 3:** Predictors of hospitalization costs of patients with stroke.

Variable	Standardized coefficient	Adjusted Std. Err.	95% CI		*p*-value
Gender					
Male	0.007	444.303	−182.151	1559.543	0.121
Female	Reference				
Age group					
<45	0.015	626.389	737.193	3192.674	0.002
45–64	−0.009	446.012	−1685.915	62.479	0.069
≥65	Reference				
Type of stroke					
ICH	Reference				
SAH	0.134	1817.412	54776.897	61901.257	0.000
IS	−0.087	473.336	−9278.242	−7422.737	0.000
TIA	−0.097	728.831	−15818.263	−12961.202	0.000
Hospital level					
Unclassified	−0.263	840.790	−52494.792	−49198.845	0.000
Primary hospital	−0.120	1006.745	−25110.963	−21164.462	0.000
Secondary hospital	−0.129	611.231	−19173.248	−16777.185	0.000
Tertiary hospital	Reference				
Hospital type					
Public hospital	−0.003	841.276	−2109.210	1188.643	0.000
Private hospital	Reference				
Admission year					
2019	−0.036	1075.122	−11163.804	−6949.262	0.000
2020	−0.018	1066.373	−6625.226	−2444.980	0.000
2021	−0.010	401.434	−1730.330	−156.685	0.019
2022	Reference				
LOS	0.632	7.617	944.595	974.453	0.000
Adjusted R^2^, % (N:35999)	40.1	36147.250	–	–	0.000

**Figure 2:**
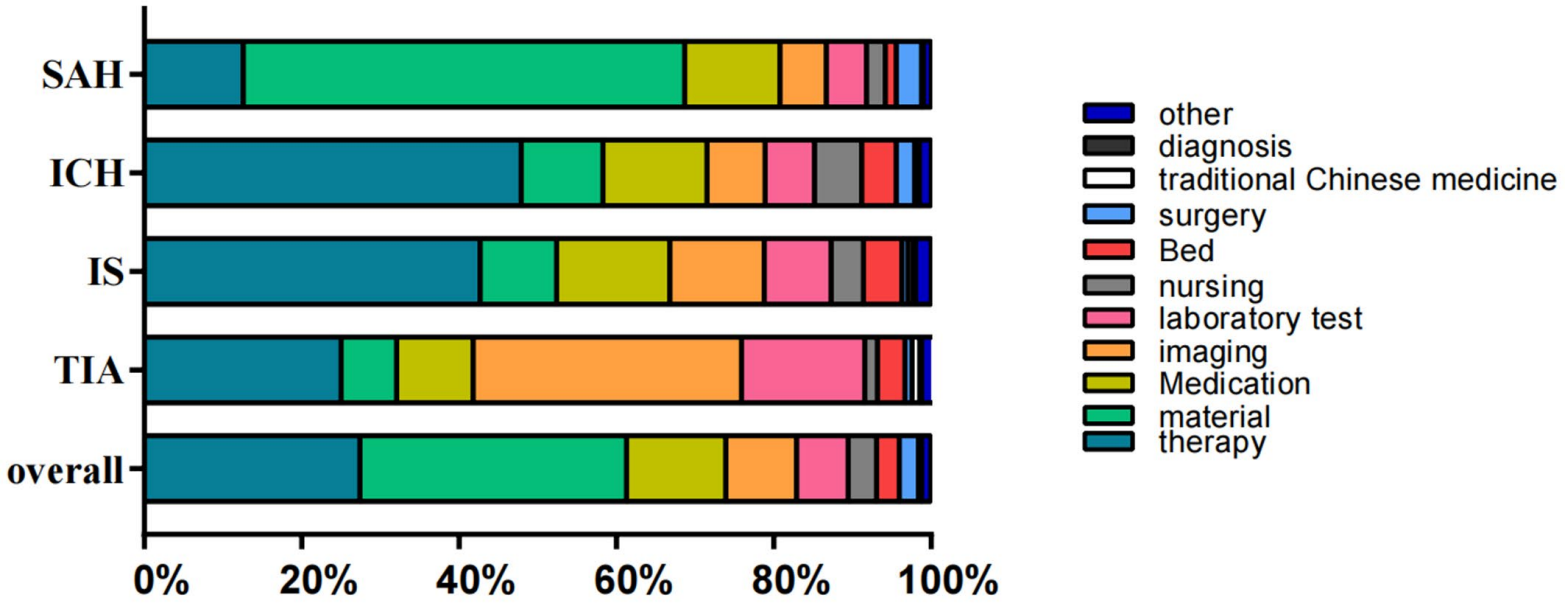
The composition of hospitalization cost (in percentage). SAH, subarachnoid hemorrhage; ICH, intracerebral hemorrhage; IS, ischemic stroke; TIA, transient ischemic attack.

The largest portion of hospitalization costs came from material consumption, which constituted 33.9% of patients’ total expenses. This was followed by rehabilitation therapy (27.4%), medication expenses (12.6%) and imaging costs (9.0%). Medical service fees, including diagnosis, surgery, and nursing, made up only 6.1% of expenditures in total. (6.1%). When classifying costs based on stroke subtypes, materials accounted for a larger share of costs in SAH patients (56.1%). Additionally, patients with ICH and IS incurred higher costs for drugs and rehabilitation therapy, whereas those with TIA spent more on examinations and laboratory fees (Figure 2).

## 4 Discussion

In China, the burden of stroke is anticipated to escalate due to population aging and a persistent high prevalence of stroke risk factors. Nevertheless, comprehensive analysis of the costs associated with different stroke subtypes has been lacking. Delineating the distribution and attributes of hospitalization costs for stroke patients and investigating the contributing factors are vital to delivering high-quality healthcare services while addressing the growing socioeconomic burden. This study conducted a comprehensive assessment of the distribution of stroke-related hospitalization costs in southeastern China. Our findings underscore the substantial economic burden placed on both individuals and society by stroke, with significant variation in economic impact observed among different stroke subtypes.

### 4.1 Distribution of patient characteristics

Notably, the number of stroke cases, regardless of the type, surged in 2020. Since the onset of the coronavirus disease 2019 (COVID-19) pandemic, a considerable proportion of patients with COVID-19 have experienced thrombotic complications and stroke (14). Increasing evidence suggests a potential association between COVID-19 infection or COVID-19 vaccination and stroke. In a prior study conducted in Wuhan, China, it was revealed that 2.3% of 214 hospitalized COVID-19 patients had previously experienced an ischemic stroke (15). A number of other studies have also documented cases of stroke in COVID-19 patients, with incidence rates similar to those observed in the early stages of the epidemic in Wuhan, China (16–18). Sadeghmousavi et al. explored stroke as a potential complication of COVID-19 and its potential mechanisms of harm (19), de Mélo Silva Jr. et al. suggested the potential relationship between COVID-19 vaccination and the risk of cerebral hemorrhage (20), and an ischemic stroke associated with the COVID-19 vaccine was reported by Famularo et al. (21).

Epidemiological data suggest that stroke is a sexually dimorphic disease affecting men more than women (22). Among our study participants, more than half were male. The evidence has accumulated demonstrating that estrogen, particularly 17-hydroxystanol, is protective for the brain and reduces stroke risks in women (23, 24). Traditionally, stroke has been viewed as a disease of the older adult; however, our data indicate that the average age of onset for various types of stroke is below 60 years, with the youngest age being 1 year old. It is reported that the global incidence and hospitalization rate of stroke among young people are rising, with young adults (<50 years old) accounting for 10%–14% of all strokes. Additionally, data from the Global Burden of Disease (GBD) indicates a significant rise in global childhood stroke incidence since 1990. Between 1990 and 2013, the absolute number of childhood strokes worldwide increased by 1.5 times. As a result, stroke among young people and children is becoming an increasingly serious issue in both developing and developed countries (25–29).

Our study revealed that patients with stroke exhibited a greater propensity to seek medical care at tertiary hospitals or public healthcare facilities. Tertiary public hospitals are likely to be preferred because of their specialized expertise in managing complex medical conditions with highly trained specialists. Advanced medical technology and facilities that offer accurate diagnoses and innovative treatments attract patients seeking cutting-edge care. Comprehensive services and strong reputations for delivering high-quality care increasingly make tertiary hospitals the preferred choice for patients seeking medical treatment. However, rehabilitation hospitalization expenses in tertiary hospitals were found to be higher than those in other medical institutions after adjusting for confounders, which accords with other study findings (30). Another study conducted among 4,716 immobile patients with stroke in 25 hospitals across six provinces in China revealed that the hospitalization expenses of immobile patients in tertiary hospitals were higher than those in non-tertiary hospitals (31). A further study in northeast China enrolled 138,757 patients from 39 hospitals and revealed that rehabilitation hospitalization expenses in tertiary hospitals (CNY 10,046.9/USD 1493.1) were higher than those in secondary hospitals (CNY6286.6/USD 934.7) (10). This may be partially explained by the unequal distribution of health resources and services in China. In the hierarchical medical system, patients with minor illnesses are generally directed to primary medical institutions, while those with serious illnesses are referred to specialized medical facilities for diagnosis and treatment. Afterward, they are advised to return to primary medical institutions for postoperative care. Implementing appropriate hierarchical medical system policies is crucial because such policies optimize healthcare delivery by guiding patients to the most suitable care level based on their medical condition, thereby enhancing treatment effectiveness and resource utilization (32).

### 4.2 Regional differences in costs

Stroke hospitalization costs in our study were estimated at CNY 31,339.4/USD 4659.4, which was higher than those reported in other Chinese regions (5, 10, 33, 34). Economic development as well as the quality of medical services may contribute to this discrepancy. Guangdong province’s gross domestic product (GDP) stood at the highest level among 31 provinces and autonomous regions in China in 2017, according to the National Bureau of Statistics (35). Therefore, our reported costs may be higher than average national costs, especially as the estimated costs for ICH (CNY 48,724.2/USD 7244.0) and IS (CNY 26,550.3/USD 3947.4) in our study were above the national average costs (CNY 26,087.9/USD 3878.6 for ICH and CNY 10,740.7/USD 1596.9 for IS) reported in the 2022 China Health Statistical Yearbook. However, our reported costs were lower than those for developed countries in Europe, Japan and North America. A United States (U.S.) study suggested that the average cost of stroke was USD 20,396 (± 23,256), which is equivalent to CNY 141,652.26 (± 161,515.25) (36). Research from Croatia indicates that the average hospitalization cost for ischemic types of stroke was Euro (€)18,221 (equivalent to CNY 142,486.40) (37), while a study in Germany showed that the average cost for hemorrhagic stroke was $26,602 (38). Tu et al. revealed that the average (median) hospitalization charges per stroke patient in Fukuoka, Japan were $9,020 ($7,974), ranging from $336 to $54,509 (39).

### 4.3 Costs and stroke type

Different types of stroke exhibit distinct clinical features and management requirements, which significantly affect hospitalization costs. SAH patients may experience a sudden severe headache and require comprehensive evaluation through imaging studies and possible surgical repair. The risk of complications, particularly vasospasm, necessitates extended monitoring and management in an intensive care setting, thereby escalating hospitalization costs. The management of ICH often requires urgent neurosurgical interventions, intensive monitoring, and rehabilitation services, leading to prolonged hospital stays and higher costs due to potential complications like rebleeding, hydrocephalus, or infection. The severity of IS can vary based on the size and location of the infarct. Treatment may involve thrombolysis, thrombectomy, and subsequent rehabilitation. While initial treatment costs can be substantial, timely intervention may reduce the length of hospitalization and long-term care needs, impacting overall costs positively. Nevertheless, post-stroke complications, such as pneumonia or deep vein thrombosis, can increase expenses significantly. Symptoms of TIA resolve within 24 hours, leading to shorter hospital stays and generally lower treatment costs compared to the other conditions (40, 41).

In our study, the mean hospitalization costs for hemorrhagic stroke (CNY 93,454.9/USD 13894.4 for SAH and CNY 48,724.2/USD 7244.0 for ICH) were notably higher than those for ischemic types of stroke (CNY 26,550.3/USD 3947.4 for IS and CNY 11,170.1/USD 1660.7 for TIA), which is consistent with previous studies (10, 31). It is likely that this is due to the frequent requirement for surgery and intensive care monitoring, along with the high levels of morbidity that follow. Hemorrhagic stroke typically demands more intricate medical treatments or nursing interventions compared to ischemic strokes. Therefore, the median cost of materials, medical services, and total expenses for patients with hemorrhagic stroke may be higher (42). According to our results, a more extensive surgical treatment was also given to patients with hemorrhagic stroke than to those with ischemic strokes, according to our results (Table 1), and that they also spent more on surgery than those with ischemic types of stroke (Figure 2). Ischemic types of stroke are more common, while hemorrhagic types of stroke account for a higher number of deaths and DALYs (42). Hence, it is essential to enhance the medical insurance reimbursement ratio for hemorrhagic stroke patients in order to alleviate the cost of personal medical care.

### 4.4 Hospitalization cost categories

The cost composition analysis revealed that material fees represented the largest proportion of overall hospitalization costs (as high as 33.90%); therapy fees (27.40%) and medication fees (12.60%) ranked second and third in terms of total hospitalization costs, respectively. As indicated by the analysis of the cost composition, material expenses accounted for the largest proportion of the total cost of hospitalization (33.90%). The second and third highest costs were therapy fees (27.40%) and medication fees (12.60%). While the cost composition largely aligns with previous study findings, the proportion of medication fees appears to be lower (3, 43, 44). This may be attributed to significant reforms in China’s health system implemented in public hospitals over the past decade, which eliminated markups on expenses of medications and promoted the use of more affordable drugs (45, 46). When classifying costs based on stroke subtypes, SAH requires surgical intervention performed by endovascular coiling or surgical clipping to prevent rebleeding from the same aneurysm, resulting in the highest proportion of material costs in hospitalization expenses. Patients with ICH and IS often experience multiple functional impairments, necessitating more intensive rehabilitation interventions, which usually require multiple examinations to rule out organic lesions before making a diagnosis. Therefore, adjusting cost categories based on different stroke subtypes may be a strategy to lower charges for hospitals and alleviate the China’s stroke economic burden.

### 4.5 Correlation of hospitalization expenses

In the linear regression analysis conducted in this study, LOS, stroke subtype (hemorrhagic stroke) hospital level and type were found to be significantly affected hospitalization costs among immobile stroke patients. By identifying these factors, we can get a better understanding of hospitalization expenses’ nature and optimize the efficiency of healthcare delivery. LOS exhibited a strong correlation with hospitalization costs among immobile stroke patients, consistent with the findings of numerous previous studies (5, 7, 42). Ma et al. reported that stroke severity, medical insurance status, comorbidities, and increased leukocyte count upon admission may serve as predictive factors for LOS of stroke patients (47). Patients in state-funded healthcare could have unnecessary LOS shortened to enhance resource allocation and cost efficiency, while other contributing factors, such as hospital level and type, could be considered in relation to the severity of the illness as well as the patient’s inclination.

This study had some limitations. First, the analysis only reported inpatient cost data, excluding outpatient expenditures and follow-up costs, which are further crucial components for accurately predicting the economic burdens faced by patients. Additional research is needed to estimate these costs and to gain a comprehensive understanding of the total expenses incurred by immobile stroke patients. Second, we were not able to establish causality or determine the direction of the observed relationships because this was a cross-sectional study. Third, this study is limited to southeastern China and during COVID-19 epidemic, whereby both the COVID and vaccination may have had a big impact on strokes. This study does not therefore represent a strokes alone study to be replicated elsewhere. Additionally, stroke severity information was not available in the accessed database, which is likely to be a key factor influencing service costs. Potential risk factors, comorbidities, and patient prognoses were excluded from the database. We intend to conduct deeper and more wide-ranging investigations in future.

The advantage of this study lies in analyzing the distribution of hospitalization costs and predictors of them for various stroke subtypes. The patients enrolled in this prospective multicenter study were from 50 units in Shenzhen. We identified important factors influencing health care costs through analysis of hospitalization expenses sources. This identification is likely to facilitate the implementation of effective measures aimed at reducing costs for individuals, society, and the government. Therefore, our findings hold significance in terms of economic assessments aimed at supporting policymaking regarding reimbursements, investment strategies, and pricing structures for medical and nursing interventions. This study has practicality in developing countries as well as in developed countries aspiring to offer the full spectrum of healthcare services for stroke patients and to achieve a better balance between their personal costs, health benefits, and social welfare expenses.

## 5 Conclusion

Stroke imposes an increasingly heavy financial burden on both patients and society in Guangdong Province, southeastern China, especially after the COVID-19 pandemic. The distribution of hospitalized patients in this study varies in terms of admission time, gender, and hospital type. Our results indicated that tertiary public hospitals were under immense medical pressure, as most patients with stroke sought treatment at these facilities. However, rehabilitation hospitalization expenses in tertiary hospitals were found to be higher than those in other types of medical institutions. Differences were found in terms of the burden among different types of stroke, with hemorrhagic stroke resulting in greater hospitalization costs, whereas ischemic types of stroke had a greater incidence rate, and the proportion of hospitalization cost categories varied among different types of stroke. Additionally, the LOS, the type and level of hospital significantly affected hospitalization costs in immobile stroke patients with hemorrhagic as well as ischemic stroke types. Reducing the economic burden of stroke in China is most likely achieved by implementing hierarchical medical system policies, increasing the reimbursement ratio of medical insurance for hemorrhagic stroke, tailoring cost categories to accommodate varying stroke subtypes, shortening LOS, and taking comprehensive measures to control other related factors.

## Data Availability

The original contributions presented in the study are included in the article/Supplementary material, further inquiries can be directed to the corresponding author.
