# Supplementary material for: Hospitalization costs in patients with stroke in southeastern China: a retrospective population-based cohort study, 2019–2022
Source: Front Public Health. 2024 Nov 8;12:1442171. doi: 10.3389/fpubh.2024.1442171 (PMC11582024; doi:10.3389/fpubh.2024.1442171)
Supplement: Supplementary file 1 [file Data_Sheet_1.docx]

**Supplemental table 1-1. Correlation coefficients of continuous variables and ordinal categorical variables**

|  | Cost | LOS | hospital level | age group | admission year |
| --- | --- | --- | --- | --- | --- |
| Cost | 1 |  |  |  |  |
| LOS | 0.537^***^ | 1 |  |  |  |
| hospital level | 0.085^***^ | -0.357^***^ | 1 |  |  |
| age group | -0.048^***^ | 0.129^***^ | -0.158^***^ | 1 |  |
| admission year | -0.167^***^ | -0.239^***^ | 0.121^***^ | -0.035^***^ | 1 |

***: *P*＜0.001

**Supplemental table 1-2. Chi-square test between type of stroke and gender**

|  | Value | df | Asymptotic Significance (2-sided) |
| --- | --- | --- | --- |
| Pearson Chi-Square | 1176.253^a^ | 3 | <0.001 |
| Likelihood Ratio | 1082.479 | 3 | <0.001 |
| Linear-by-Linear Association | 630.172 | 1 | <0.001 |
| N of Valid Cases | 35999 |  |  |

1. 0 cells (0.0%) have expected count less than 5. The minimum expected count is 115.85.

**Supplemental table 1-3. Chi-square test between hospital type and gender**

|  | Value | df | Asymptotic Significance (2-sided) | Exact Sig (2-sided) | Exact Sig (1-sided) |
| --- | --- | --- | --- | --- | --- |
| Pearson Chi-Square | 0.018^a^ | 1 | 0.892 |  |  |
| Continuity Correction^b^ | 0.013 | 1 | 0.908 |  |  |
| Likelihood Ratio | 0.018 | 1 | 0.892 |  |  |
| Fisher’s Exact Test |  |  |  | 0.891 | 0.454 |
| Linear-by-Linear Association | 0.018 | 1 | 0.892 |  |  |
| N of Valid Cases | 35999 | - | - |  |  |

a. 0 cells (0.0%) have expected count less than 5. The minimum expected count is 979.57.

b. Computed only for a 2x2 table

**Supplemental table 1-4. Chi-square test between hospital type and type of stroke**

|  | Value | df | Asymptotic Significance (2-sided) |
| --- | --- | --- | --- |
| Pearson Chi-Square | 744.138^a^ | 3 | <0.001 |
| Likelihood Ratio | 754.576 | 3 | <0.001 |
| Linear-by-Linear Association | 590.853 | 1 | <0.001 |
| N of Valid Cases | 35999 |  |  |

1. 0 cells (0.0%) have expected count less than 5. The minimum expected count is 40.84.

**Supplemental table 1-5. Correlation coefficients of continuous variables and binary variables**

|  | Cost | LOS | gender | hospital type |
| --- | --- | --- | --- | --- |
| Cost | 1 |  |  |  |
| LOS | 0.537^***^ | 1 |  |  |
| gender | -0.015^**^ | 0.031^***^ | 1 |  |
| hospital type | 0.191^***^ | 0.387^***^ | 0.891 | 1 |

**: *P*＜0.01, ***: *P*＜0.001

**Supplemental table 1-6. Correlation coefficients of ordinal categorical variables and binary variables**

|  | gender | hospital type | hospital level | age group | admission year |
| --- | --- | --- | --- | --- | --- |
| gender | 1 |  |  |  |  |
| hospital type | 0.001 | 1 |  |  |  |
| hospital level | -0.056^***^ | -0.318^***^ | 1 |  |  |
| age group | 0.203^***^ | 0.023^***^ | -0.158^***^ | 1 |  |
| admission year | 0.001 | -0.210^***^ | 0.121^***^ | -0.035^***^ | 1 |

***: *P*＜0.001
