# Supplementary material for: Hospitalization costs in patients with stroke in southeastern China: a retrospective population-based cohort study, 2019–2022
Source: Front Public Health. 2024 Nov 8;12:1442171. doi: 10.3389/fpubh.2024.1442171 (PMC11582024; doi:10.3389/fpubh.2024.1442171)
Supplement: Supplementary file 2 [file Data_Sheet_2.docx]

**Supplemental table 2. Predictors of hospitalization costs of patients with stroke in Unclassified** **hospital**

| Variable | Standardized Coefficient | Adjusted Std. Err. | 95% CI | | P-value |
| --- | --- | --- | --- | --- | --- |
| Gender |  |  |  |  |  |
| Male | -0.016 | 303.908 | -833.788 | 358.155 | 0.434 |
| Female | Reference |  |  |  |  |
| Age group |  |  |  |  |  |
| ＜45 | 0.234 | 744.761 | 6696.112 | 9617.111 | 0.000 |
| 45-64 | 0.172 | 356.908 | 2065.113 | 3464.928 | 0.000 |
| ≥65 | Reference |  |  |  |  |
| Type of stroke |  |  |  |  |  |
| ICH | -0.072 | 463.009 | -2493.216 | -677.268 | 0.001 |
| SAH | 0.089 | 2535.050 | 6297.302 | 16239.917 | 0.000 |
| IS | Reference |  |  |  |  |
| TIA | 0.010 | 1002.013 | -1480.738 | 2449.219 | 0.629 |
| Hospital type |  |  |  |  |  |
| public hospital | -0.153 | 740.339 | -6946.643 | -4042.990 | 0.000 |
| private hospital | Reference |  |  |  |  |
| Admission year |  |  |  |  |  |
| 2019 | - | - | - | - | - |
| 2020 | - | - | - | - | - |
| 2021 | Reference |  |  |  |  |
| 2022 | 0.058 | 287.254 | 255.195 | 1381.823 | 0.004 |
| LOS | 0.047 | 4.763 | 1.142 | 19.823 | 0.028 |
| Adjusted R^2^, %  (N:2237) | 10.3 | 6672.066 | - | - | 0.000 |

**Supplemental table 3. Predictors of hospitalization costs of patients with stroke in Primary** **hospital**

| Variable | Standardized Coefficient | Adjusted Std. Err. | 95% CI | | P-value |
| --- | --- | --- | --- | --- | --- |
| Gender |  |  |  |  |  |
| Male | 0.056 | 11069.676 | 3283.526 | 7478.843 | 0.000 |
| Female | Reference |  |  |  |  |
| Age group |  |  |  |  |  |
| ＜45 | -0.073 | 1705.082 | -13753.188 | -7065.783 | 0.000 |
| 45-64 | -0.054 | 1102.033 | -7065.551 | -2743.330 | 0.000 |
| ≥65 | Reference |  |  |  |  |
| Type of stroke |  |  |  |  |  |
| ICH | 0.021 | 1117.936 | -217.871 | 4166.722 | 0.078 |
| SAH | -0.003 | 6918.178 | -1562.095 | 11671.293 | 0.000 |
| IS | Reference |  |  |  |  |
| TIA | 0.002 | 2339.577 | -4243.311 | 49332.609 | 0.883 |
| Hospital type |  |  |  |  |  |
| public hospital | -0.043 | 1296.891 | -7213.035 | -2126.573 | 0.000 |
| private hospital | Reference |  |  |  |  |
| Admission year |  |  |  |  |  |
| 2019 | 0.037 | 1492.719 | 1688.283 | 7542.791- | 0.002 |
| 2020 | 0.098 | 1489.310 | 8986.511 | 14827.647 | 0.000 |
| 2021 | Reference |  |  |  |  |
| 2022 | -0.039 | 1214.135 | -6338.006 | -1576.117 | 0.001 |
| LOS | 0.783 | 10.516 | 638.345 | 689.587 | 0.000 |
| Adjusted R^2^, %  (N:2257) | 74.5 | 22749.519 | - | - | 0.000 |

**Supplemental table 4. Predictors of hospitalization costs of patients with stroke in Secondary hospital**

| Variable | Standardized Coefficient | Adjusted Std. Err. | 95% CI | | P-value |
| --- | --- | --- | --- | --- | --- |
| Gender |  |  |  |  |  |
| Male | 0.001 | 877.160 | -1630.752 | 1808.548 | 0.919 |
| Female | Reference |  |  |  |  |
| Age group |  |  |  |  |  |
| ＜45 | -0.011 | 1267.268 | -3800.380 | 1168.513 | 0.299 |
| 45-64 | -0.042 | 880.864 | -5095.493 | -1641.668 | 0.000 |
| ≥65 | Reference |  |  |  |  |
| Type of stroke |  |  |  |  |  |
| ICH | Reference |  |  |  |  |
| SAH | 0.120 | 5289.812 | 57020.626 | 77761.715 | 0.000 |
| IS | -0.037 | 848.525 | -4642.555 | -1315.530 | 0.000 |
| TIA | -0.026 | 1838.226 | -8320.714 | -1113.120 | 0.010 |
| Hospital type |  |  |  |  |  |
| public hospital | 0.032 | 1290.711 | 1788.053 | 6848.867 | 0.001 |
| private hospital | Reference |  |  |  |  |
| Admission year |  |  |  |  |  |
| 2019 | -0.001 | 1644.933 | -3315.242 | 3134.457 | 0.569 |
| 2020 | 0.035 | 1530.338 | 2523.574 | 8523.955 | 0.000 |
| 2021 | Reference |  |  |  |  |
| 2022 | -0.031 | 826.902 | -4175.036 | -932.794 | 0.002 |
| LOS | 0.744 | 10.700 | 752.057 | 794.011 | 0.000 |
| Adjusted R^2^, %  (N:4655) | 59.2 | 25626.567 | - | - | 0.000 |

**Supplemental table 5. Predictors of hospitalization costs of patients with stroke in Tertiary hospital**

| Variable | Standardized Coefficient | Adjusted Std. Err. | 95% CI | | P-value |
| --- | --- | --- | --- | --- | --- |
| Gender |  |  |  |  |  |
| Male | 0.000 | 535.884 | -1090.926 | 1009.793 | 0.940 |
| Female | Reference |  |  |  |  |
| Age group |  |  |  |  |  |
| ＜45 | 0.028 | 725.857 | 2284.358 | 5129.793 | 0.000 |
| 45-64 | -0.002 | 531.477 | -1190.867 | 892.578 | 0.779 |
| ≥65 | Reference |  |  |  |  |
| Type of stroke |  |  |  |  |  |
| ICH | Reference |  |  |  |  |
| SAH | 0.149 | 1980.259 | 57910.284 | 65673.103 | 0.000 |
| IS | -0.069 | 573.091 | -7993.140 | -5746.565 | 0.000 |
| TIA | -0.072 | 827.831 | -111833.915 | -8588.731 | 0.000 |
| Hospital type |  |  |  |  |  |
| public hospital | 0.081 | 1202.594 | 16695.884 | 21410.178 | 0.000 |
| private hospital | Reference |  |  |  |  |
| Admission year |  |  |  |  |  |
| 2019 | -0.043 | 1480.589 | -16341.164 | -10537.103 | 0.000 |
| 2020 | -0.036 | 1508.437 | -14275.153 | -8361.923 | 0.000 |
| 2021 | -0.022 | 470.884 | -3032.125 | -1186.212 | 0.000 |
| 2022 | Reference |  |  |  |  |
| LOS | 0.634 | 12.557 | 1466.731 | 1515.956 | 0.000 |
| Adjusted R^2^, %  (N:26850) | 41.9 | 37143.019 | - | - | 0.000 |
